# Effects of Vascular Comorbidity on Cognition in Multiple Sclerosis Are Partially Mediated by Changes in Brain Structure

**DOI:** 10.3389/fneur.2022.910014

**Published:** 2022-05-24

**Authors:** Ruth Ann Marrie, Ronak Patel, Chase R. Figley, Jennifer Kornelsen, James M. Bolton, Lesley A. Graff, Erin L. Mazerolle, Carl Helmick, Md Nasir Uddin, Teresa D. Figley, James J. Marriott, Charles N. Bernstein, John D. Fisk

**Affiliations:** ^1^Department of Internal Medicine, Max Rady College of Medicine, Rady Faculty of Health Sciences, University of Manitoba, Winnipeg, MB, Canada; ^2^Department of Community Health Sciences, Max Rady College of Medicine, Rady Faculty of Health Sciences, University of Manitoba, Winnipeg, MB, Canada; ^3^Department of Clinical Health Psychology, Max Rady College of Medicine, Rady Faculty of Health Sciences, University of Manitoba, Winnipeg, MB, Canada; ^4^Department of Radiology, Max Rady College of Medicine, Rady Faculty of Health Sciences, University of Manitoba, Winnipeg, MB, Canada; ^5^Division of Diagnostic Imaging, Winnipeg Health Sciences Centre, Winnipeg, MB, Canada; ^6^Neuroscience Research Program, Kleysen Institute for Advanced Medicine, Winnipeg Health Sciences Centre, Winnipeg, MB, Canada; ^7^Department of Psychiatry, Max Rady College of Medicine, Rady Faculty of Health Sciences, University of Manitoba, Winnipeg, MB, Canada; ^8^Department of Psychology, St. Francis Xavier University, Antigonish, NS, Canada; ^9^Department of Psychiatry and Division of Geriatric Medicine, Dalhousie University, Halifax, NS, Canada; ^10^Department of Neurology, University of Rochester, Rochester, New York, NY, United States; ^11^Nova Scotia Health and the Departments of Psychiatry, Psychology & Neuroscience, and Medicine, Dalhousie University, Halifax, NS, Canada

**Keywords:** multiple sclerosis, MRI, cognition, diabetes, hypertension

## Abstract

**Objective:**

Vascular comorbidities are associated with reduced cognitive performance and with changes in brain structure in people with multiple sclerosis (MS). Understanding causal pathways is necessary to support the design of interventions to mitigate the impacts of comorbidities, and to monitor their effectiveness. We assessed the inter-relationships among vascular comorbidity, cognition and brain structure in people with MS.

**Methods:**

Adults with neurologist-confirmed MS reported comorbidities, and underwent assessment of their blood pressure, HbA1c, and cognitive functioning (i.e., Symbol Digit Modalities Test, California Verbal Learning Test, Brief Visuospatial Memory Test-Revised, and verbal fluency). Test scores were converted to age-, sex-, and education-adjusted z-scores. Whole brain magnetic resonance imaging (MRI) was completed, from which measures of thalamic and hippocampal volumes, and mean diffusivity of gray matter and normal-appearing white matter were converted to age and sex-adjusted z-scores. Canonical correlation analysis was used to identify linear combinations of cognitive measures (cognitive variate) and MRI measures (MRI variate) that accounted for the most correlation between the cognitive and MRI measures. Regression analyses were used to test whether MRI measures mediated the relationships between the number of vascular comorbidities and cognition measures.

**Results:**

Of 105 participants, most were women (84.8%) with a mean (SD) age of 51.8 (12.8) years and age of symptom onset of 29.4 (10.5) years. Vascular comorbidity was common, with 35.2% of participants reporting one, 15.2% reporting two, and 8.6% reporting three or more. Canonical correlation analysis of the cognitive and MRI variables identified one pair of variates (Pillai's trace = 0.45, *p* = 0.0035). The biggest contributors to the cognitive variate were the SDMT and CVLT-II, and to the MRI variate were gray matter MD and thalamic volume. The correlation between cognitive and MRI variates was 0.50; these variates were used in regression analyses. On regression analysis, vascular comorbidity was associated with the MRI variate, and with the cognitive variate. After adjusting for the MRI variate, vascular comorbidity was not associated with the cognitive variate.

**Conclusion:**

Vascular comorbidity is associated with lower cognitive function in people with MS and this association is partially mediated via changes in brain macrostructure and microstructure.

## Introduction

Multiple sclerosis (MS) is a central nervous system disease characterized by multiple signs and symptoms, including cognitive impairment. Over 40% of individuals with MS struggle with cognitive impairment (1, 2) and its adverse effects on daily function (3). MS is characterized by demyelination and axonal injury, therefore it is associated with macrostructural changes in the brain such as atrophy, as well as microstructural changes in normal appearing white matter (NAWM). Lower whole brain and regional gray matter volumes, particularly thalamic volumes (4) are associated with cognitive dysfunction (5–7). Microstructural abnormalities, as measured using diffusion tensor imaging (DTI) appear to provide even stronger prediction of cognitive impairment than macrostructural abnormalities (8–10).

Comorbid conditions are highly prevalent among individuals with MS (11). The vascular comorbidities of hypertension and hyperlipidemia are among the most common comorbidities with MS, and increase in prevalence with age. They are associated with outcomes such as relapses, disability progression and lower quality of life (12, 13). More recent studies suggest that hypertension and diabetes are also associated with reduced cognitive function in domains such as processing speed, verbal learning and visual memory for persons with MS (14–16). However, findings have varied across studies, possibly reflecting differences in study populations, comorbidity measurement and cognitive tests employed. Although findings are inconsistent as to the magnitude of the effect and the specific comorbidities involved (14, 17–19), vascular comorbidities have been associated with macrostructural brain changes such as lower brain volumes in people with MS. In the general population widespread changes in white matter microstructure are known to be associated with vascular risk factors; mean diffusivity (MD) appears to be more sensitive to these effects than FA or mean kurtosis (20, 21). The association of vascular comorbidity and brain microstructure has not been explored in people with MS.

Depression and anxiety disorders are other common comorbidities associated with lower cognitive performance in people with MS (22). Depression has also been associated with lower brain volumes, specifically affecting the temporal lobes and hippocampus (23–25), and has also been associated with altered microstructure in the form of higher MD in NAWM and gray matter in the left temporal lobe in persons with MS (26). Therefore these comorbidities need to be accounted for when the effects of vascular comorbidities on brain structure and cognition are assessed.

Better understanding of the relationships among comorbidities, brain structural changes, and outcomes such as cognitive functioning is needed for persons with MS. Understanding causal pathways is necessary to support the design of interventions to mitigate the impacts of comorbidities, and to monitor their effectiveness. For example, if the effects of vascular comorbidity on cognition were mediated by changes in brain structure, intervention studies aimed at treating vascular comorbidity to improve cognition could use MRI measures as intermediate outcomes to enable shorter, smaller studies. We aimed to extend our prior work examining relations between comorbidity and cognition (15) and hypothesized that the effects of vascular comorbidity on cognition would be mediated by changes in brain structure in people with MS.

## Methods

### Study Population

As described previously (15), our study sample was drawn from a subgroup of adults with MS participating in a longitudinal study regarding psychiatric comorbidity in immune-mediated inflammatory diseases (the “IMID” study). This subgroup included persons aged ≥18 years with definite MS (27), as confirmed by a neurologist and medical records review. Exclusion criteria included comorbid brain tumors, neurodegenerative disorders, or contraindications to MRI. We did not exclude any other comorbidities because comorbidities (predominantly vascular and psychiatric) were the focus of the sub-study.

We also enrolled healthy controls who have been described in detail elsewhere (28). Briefly, healthy controls were aged 18 years or older. Exclusion criteria for this group included any chronic medical condition including vascular comorbidities, cognitive impairment, a positive response to the Structured Clinical Interview for DSM-IV (SCID-IV) screening questions for depressive or anxiety disorders, head injury associated with loss of consciousness or amnesia, or chronic medication use (29). Hypertension, as measured during the study visit, was also an exclusion criterion. For this analysis, they predominantly served to allow us to develop regression-based norms for cognitive and MRI measures.

All participants in the sub-study underwent standardized assessments of physical, cognitive, and mental health functioning, which they completed the same day. They also had a brain MRI, which was completed within a maximum of 4 weeks of the study visit in which they completed their standardized assessments (30). All participants provided written informed consent. The University of Manitoba Health Research Ethics Board approved the study. Study data were collected and managed using REDCap electronic data capture tools (31) hosted at the University of Manitoba.

### Sociodemographic Information

Participants reported gender, date of birth, race and ethnicity, highest level of education attained, annual household income, and marital status using self-administered questionnaires. Race and ethnicity were assessed using response options from Statistics Canada; race was categorized as white vs. non-white because the number of non-white participants was too small to further subdivide. We categorized level of education as high school or less, vs. more than high school (including college, university, technical/trade).

### Clinical Characteristics

Age at MS symptom onset, clinical course (relapsing remitting, secondary progressive, primary progressive), relapses in the last 12 months, and current disease-modifying therapy (DMT) were determined based on patient report and medical records review. The Expanded Disability Status Scale (EDSS) was assessed by a certified neurologist (RAM/JJM) (32).

### Comorbidity and Health Behaviors

Participants reported their lifetime history of comorbidities (including hypertension, diabetes, hyperlipidemia, and heart disease) using a validated questionnaire (33), including the year of diagnosis and whether the condition was currently treated. This information was complemented by medical records review and other assessments (15). During the study visit, we recorded blood pressure in the seated position using an automatic blood pressure machine. Participants were classified as currently having hypertension if they reported physician-diagnosed hypertension, or had an elevated blood pressure of at least 140/90 mm Hg, and/or used anti-hypertensive medications. Participants were classified as currently having diabetes if they self-reported physician-diagnosed diabetes, used medications for diabetes and/or had a hemoglobin A1c measured at the study visit >6.5% (34). We did not discriminate between type 1 and type 2 diabetes. Participants were classified as currently having heart disease if they self-reported physician-diagnosed heart disease. We classified current smoking status as yes/no. We calculated body mass index (BMI, kg/m^2^) based on measured height and weight.

Given prior findings in the literature indicating that psychiatric comorbidity affects cognition in MS including our prior work (22, 35), current major depression and anxiety disorders were assessed for inclusion as covariates using the Structured Clinical Interview for DSM-IV (SCID-IV) (36), which was administered by trained study staff, as described elsewhere (30). We classified each condition as present or absent.

### Cognitive Function

As delineated elsewhere, we chose validated neuropsychological assessments included in the Brief International Cognitive Assessment for Multiple Sclerosis (BICAMS) (37), and which tested most cognitive domains addressed via the Minimal Assessment of Cognitive Function in MS (MACFIMS) (38). BICAMS uses the Symbol Digit Modalities Test (SDMT) (39), the California Verbal Learning Test (CVLT-II; Trial 1–5 total recall score) (40), and the Brief Visuospatial Memory Test-Revised (BVMT-R; summed recall score for all three learning trials) (41). The MACFIMS includes all of the tests from BICAMS, the Controlled Oral Word Association Test (fluency) as well as the Paced Auditory Serial Addition Test (processing speed and working memory), Delis-Kaplan Executive Function System Sorting Test (executive function), and Judgement of Line Orientation Test (spatial processing). Specifically, we used the SDMT (39) to assess information processing speed, the CVLT-II; Trial 1–5 total recall score (40) to assess verbal learning and memory, the BVMT-R (summed recall score for all three learning trials) (41) to assess visual learning and memory, and tests of verbal fluency (letter and animal categories) (42) to assess language and executive abilities. We converted raw test scores to age-, sex- and education-adjusted z-scores using local regression-based norms because we previously demonstrated that these performed better in our population than other published regression-based norms (28). *Z*-scores of ≤-1.5 were classified as impaired. To characterize the sample we also included the Wechsler Test of Adult Reading (WTAR) (43) that provided an age-, sex-, education-, and ethnicity-adjusted Full Scale IQ estimate of premorbid intelligence. Test administration was completed by trained study staff, overseen by a registered clinical neuropsychologist.

### Magnetic Resonance Imaging

#### Acquisition

As described previously (18), all participants underwent a 3 Tesla brain MRI (Siemens TIM Trio, software version VB17a, Siemens Healthcare, Erlangen, Germany; Siemens 32-channel receive-only head coil), within 4 weeks of their study visit. The images acquired included a high-resolution T1-weighted (T1w) whole brain 3D magnetization prepared rapid gradient echo (MPRAGE), dual-echo proton density-weighted (PDw), T2-weighted (T2w), fluid attenuated inversion recovery (FLAIR) images, and two 55-direction high angular resolution diffusion imaging (HARDI) scans that had phase encoding in opposite directions (see Supplementary Table e1 for scan parameters). Gadolinium was not administered. A radiologist reviewed the MRIs to screen for any clinically relevant findings unrelated to MS. All images were visually reviewed to assess for bulk motion or other artifacts.

#### T1-Weighted Images

We used FSL's FLIRT, and FNIRT (https://fsl.fmrib.ox.ac.uk/fsl/fslwiki/FLIRT) to linearly and non-linearly warp the T1w brain images to the MNI152 template (44, 45). We created lesion masks from FLAIR and T1w images using the Lesion Segmentation Tool (LST) for SPM (46), and FSL's automated Brain Intensity AbNormality Classification Algorithm (BIANCA; https://fsl.fmrib.ox.ac.uk/fsl/fslwiki/BIANCA) (47). We created final lesion masks for each participant as a binary cluster overlap of the BIANCA and LST maps, as we have found that LST is more specific but less sensitive and BIANCA is more sensitive but less specific. This allowed us to eliminate spurious small clusters identified by only one technique, reducing false positives. Lesions were filled using the lesion filling command in FSL by inputting each participant's: (1) cluster-overlapped T1w_final_lesion_map, (2) binary WM tissue map, and (3) bias-corrected T1w_brain (48). We estimated whole brain volume and gray matter volume from lesion-filled T1w images using FSL's SIENA (https://fsl.fmrib.ox.ac.uk/fsl/fslwiki/SIENA) (49, 50). Volume estimates for the thalamus (total of right and left) and hippocampus (total of right and left) were obtained using FSL FIRST (https://fsl.fmrib.ox.ac.uk/fsl/fslwiki/FIRST). All volumes were normalized relative to intracranial volume for each participant.

#### Diffusion-Weighted Images

##### Artifact Correction

Diffusion-weighted images were processed using SPM12 Artifact Correction in Diffusion MRI Toolbox (ACID; version beta 02; http://diffusiontools.com). This included simultaneous motion and eddy current correction (51), and EPI distortion correction based on the opposite polarity DWI images using the Hyperelastic Susceptibility artifact Correction (HySCo) algorithm (52, 53).

##### Tensor Model Fitting

We used the Fit Diffusion Tensor module to generate fractional anisotropy (FA), mean diffusivity (MD), radial diffusivity (RD) and axial diffusivity (AD) maps. The robust least-squares fitting algorithm was used to down-weight potential outliers in the diffusion signal (54).

##### Registration

We non-linearly warped each participant's high resolution, lesion-filled T1w image to the MNI52 Template (using the geodesic shooting method in the Computational Anatomy Toolbox for SPM12 (CAT12 version r1318; http://www.neuro.uni-jena.de/cat/)), then co-registered each of the diffusion maps to the participant's T1w image (by co-registering the b0 image and applying the same transformations). Then we spatially normalized the diffusion maps to the MNI152_T1_1mm template using subject-specific deformation fields generated previously using CAT12. We extracted mean values of these four DTI metrics for whole brain white matter (WM) as well as gray matter (GM) using each participant's CAT12 tissue segmentations, and calculated mean values for NAWM by removing voxels within each participant's lesion mask from their CAT12 WM segmentation.

##### Choice of Diffusion Metric

It is increasingly recognized that a large proportion of white matter fiber tracts have complex architecture including crossing fibers such that variations in DTI measures do not necessarily reflect variations in structural integrity of myelin or axons (55, 56). Of the four DTI measures, FA, AD, and RD are most affected by this and therefore we focused our analyses on MD (57).

#### Regression-Based Norms

Using a healthy control population which was enrolled concurrently and underwent the same study procedures, we developed regression-based norms for each MRI measure that incorporated age and gender, similar to the approach used to develop norms for cognitive tests in this population (28). This allowed us to convert each MRI measure to a z-score, enhancing their comparability despite the differences in their value ranges, and normalizing them for subsequent regression analyses. Because this was a healthy control population this means that negative z-scores for a brain volume, for example, indicated that the brain volume is lower than in a healthy person.

### Analyses

#### Descriptive

We described the study population using means (standard deviation [SD]), medians (interquartile range [IQR]), and frequencies (percent). We observed strong Spearman correlations between several of the MRI measures (Supplementary Figure e1 and Supplementary Table e2).

#### Summarizing MRI Measures

We selected 4 measures for our analyses which captured brain macrostructure and microstructure [thalamic and hippocampal volumes, MD of NAWM and of gray matter (GM)] based on as their established associations with cognition in the MS literature. These measures also met the statistical criteria of no multicollinearity amongst them (Supplementary Table e3), and met the assumption of multivariate normality required for our subsequent analyses (Supplementary Table e4).

#### Summarizing Vascular Comorbidity

Given the high degree of overlap between vascular comorbidities, and our limited sample size, we summarized the four vascular comorbidities (diabetes, hypertension, hyperlipidemia, heart disease) as a count (0, 1, 2, 3+). We used an unweighted count for consistency with a prior study showing a dose-response association between an unweighted vascular comorbidity count and brain volumes, and with performance-based measures including a cognitive test of processing speed (17). Moreover, comorbidity counts are readily understood measures that have been associated with multiple outcomes in MS (58, 59). The prior study did not include smoking or BMI in the vascular comorbidity count. Although we included smoking in the vascular count in a complementary analysis as described further below, we did not include higher BMI (i.e., being overweight or obese) in the count. Seventy-five percent of the cohort was overweight or obese. We had previously observed that higher BMI was associated with better cognitive performance (15), an effect opposite to those anticipated for other vascular comorbidities of interest on cognition, and an effect opposite to that expected on MRI outcomes. Studies in the general population suggest that higher BMI may be protective of cognition (60–62) and that this effect may be non-linear. The assumption of using an unweighted comorbidity count is that the effects of comorbidities are additive with the effects in the same direction.

#### Primary Analyses

**Figure 1:**
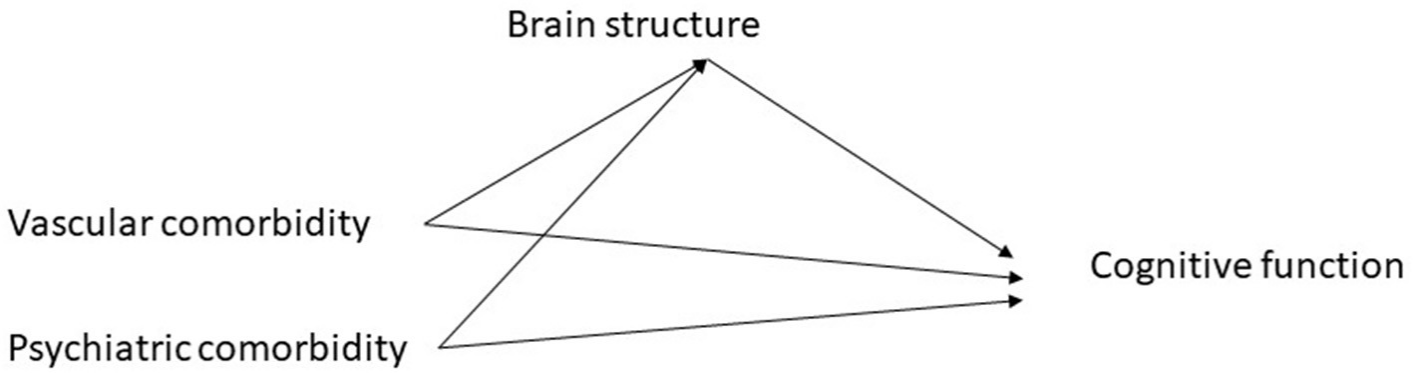
Hypothesized pathways between comorbidity and cognitive function.

Our goal was to understand the relationship between vascular comorbidity and cognition, and whether this was mediated via brain structure (Figure 1). First, a multivariate approach was used, due to the large number of variables assessing each of cognition and MRI, the size of our sample, and to minimize the number of comparisons made. Specifically, our primary analysis began with canonical correlation analysis to model the association between cognition and MRI; (63) vascular comorbidity was not evaluated in this step. Canonical correlation analysis has been used in other studies of cognition in MS (64). In this situation we view the cognitive variables as assessing a common underlying latent construct, and the MRI variables as assessing the underlying latent construct of brain structural integrity. In canonical correlation analysis, weighted linear combinations of variables (“variates”) are created within each dataset that account for the most correlation between the two datasets. The first pair of variates has the highest possible correlation, and successive pairs of variates are orthogonal and independent of other variates. Variable loadings measure the correlation between the original variable and the variate, indicating the relative contribution of the variable to the variate. This analytic approach is more powerful and reduces the number of comparisons. We assessed the assumptions of multicollinearity using correlations, multivariate normality using the Doornik-Hansen test, and linearity (Supplementary Figures e1, e2). We report the redundancy index (amount of variance explained).

Second, we constructed a series of linear regression models. In the first model, we tested the association between the count of vascular comorbidities and the cognitive variate (dependent variable). In the second model we changed the dependent variable to the MRI variate. In the third model, we tested the association between the count of vascular comorbidities and the cognitive variate (dependent variable), adjusting for the MRI variate. The count of vascular comorbidities was included as indicator variables. These regression analyses did not include age or gender since these were captured in the z-scores for the cognitive and MRI measures. In all models, covariates included current depressive disorder, current anxiety disorder and use of disease-modifying therapy (yes/no). We included use of disease-modifying therapy as a covariate because of literature suggesting that vascular comorbidity is associated with initiation (or not) of disease-modifying therapy (65), and the association of disease-modifying therapy with cognition (66). Regression analyses were bootstrapped 1,000 times, and we report bias-corrected 95% confidence intervals (95%CI). We assessed the proportion of the direct effect of comorbidity on cognition mediated by MRI as described for multi-level categorical variables (67).

#### Secondary Analyses

Third, we performed exploratory secondary analyses using multivariate regression models. These analyses aimed to provide insight into the relationships between vascular comorbidity, cognitive and MRI measures at a more granular level. However, these analyses need to be interpreted cautiously given the number of comparisons (68). We used the same three model approach as described using the canonical variates but we included the z-scores for each of the cognitive measures as dependent variables rather than the single cognitive variate, and included all of the z-scores for the MRI measures as independent variables rather than the single MRI variate. If a statistically significant global association was identified between an independent variable of interest and cognition, we explored this further using linear models which included only one cognitive z-score as the dependent variable. Non-significant global associations were not examined further.

#### Complementary Analyses

We performed complementary analyses to test the sensitivity of our findings to changes in sub-population or inclusion of other variables. First, we limited the primary analyses to women. Second, we included a history of ever smoking in the count of vascular comorbidities, and repeated the regression analyses that tested the association between the count of vascular comorbidities and the cognitive variate (dependent variable), adjusting for current depressive disorder, current anxiety disorder and use of disease-modifying therapy (yes/no) and for the MRI variate. Third, we repeated the primary analyses after limiting the study population to participants who were overweight or obese since the overlap between overweight/obesity and vascular comorbidity was too substantial to include it as a covariate.

Statistical analyses used SAS V9.4 (SAS Institute Inc., Cary, NC) and STATA 17.0 (Statacorp LLC, College Station, TX).

## Results

We included 105 participants. Most participants were women, and most had a moderate level of disability (Table 1). Vascular comorbidity was common, affecting 62 (59.0%). Just over half of participants had hypertension (50.5%), whereas only 11.4% had diabetes. Overlap between comorbidities was common. All 12 participants with diabetes had hypertension, while 10 (90.9%) were overweight or obese and 9 (75%) had hyperlipidemia. Twelve of the 53 participants with hypertension had diabetes (22.6%), while 47 (90.4%) were overweight or obese. Of six participants with heart disease, 5 (83.3%) had hypertension, and one-third had diabetes. Nearly 10% of participants currently had an anxiety disorder, of whom 8 (80%) were currently using a psychotropic medication. Fifteen percent of participants currently had a depressive disorder, of whom 13 (81.2%) were currently using a psychotropic medication. Based on average (standard error) z-scores determined using regression-based norms, cognitive performance was lowest for the SDMT (−0.76 [0.12]), followed by verbal fluency (animals, −0.61 [0.11]), verbal fluency (letter, −0.25 [0.10]), BVMT-R (−0.064 [0.11]), and the CVLT-II (0.031 [0.12]). Overall, 28 (26.7%) participants were classified as cognitively impaired based on the SDMT. In contrast, 11 (10.5%) were impaired on the CVLT-II, 13 (12.4%) on the BVMT-R, 12 (11.4%) on verbal fluency (averaging fluency for animals and letters).

**Table 1:** Cohort demographic and clinical characteristics.

**Characteristic**	**Value**
*N*	105
**Age, year** mean (SD)	51.8 (12.8)
**Female gender**, *n* (%)	89 (84.8)
**Education**, *n* (%)	
≤High School/GED	33 (32.0)
Post-secondary	70 (68.0)
**MS characteristics**	
Age at MS onset, years, mean (SD)	29.4 (10.5)
Age at MS diagnosis, years, mean (SD)	35.1 (10.2)
Disease duration, years, mean (SD)	22.4 (12.3)
**Current course**, *n* (%)	
Relapsing remitting	85 (81.7)
Secondary progressive	13 (12.5)
Primary progressive	6 (5.8)
EDSS, median (p25–p75)	3.5 (3.0–5.0)
Any relapses in last 12 months, *n* (%)	3 (2.9)
Any disease-modifying therapy, *n* (%)	58 (55.2)
Any psychotropic medication, *n* (%)	65 (61.9)
**Comorbidity & health behaviors**	
SCID Current anxiety disorder, *n* (%)	10 (9.5)
SCID Current depressive disorder, *n* (%)	16 (15.2)
Hypertension (self-reported physician diagnosis), *n* (%)	28 (26.7)
Hypertension (self-reported physician diagnosis, measured blood pressure and medication use), *n* (%)	53 (50.5)
Hyperlipidemia (self-reported physician diagnosis), *n* (%)	24 (22.9)
Hyperlipidemia (self-reported physician diagnosis and medications), *n* (%)	25 (23.8)
Diabetes (self-reported physician diagnosis), *n* (%)	11 (10.5)
Diabetes (self-reported physician diagnosis, medications and HbA1c), *n* (%)	12 (11.4)
Heart disease (self-reported physician diagnosis), *n* (%)	6 (5.7)
**No. vascular comorbidities**, ***n*** **(%)**	
0	43 (41.0)
1	37 (35.2)
2	16 (15.2)
3+	9 (8.6)
Ever smoker, *n* (%)	62 (59.1)
Current smoker, *n* (%)	15 (14.3)
BMI (kg/m^2^), mean (SD)	29.1 (6.4)

*EDSS, Expanded Disability Status Scale*.

The number of vascular comorbidities correlated with MD of NAWM (*r* = −0.27; 95%CI: −0.44, −0.086) but not with MD of GM (0.18; −0.014, 0.36), nor with thalamic (−0.084; 95%CI: −0.27, 0.11) or hippocampal (*r* = 0.001; −0.19, 0.19) volumes. Age at MS symptom onset was not correlated with the number of vascular comorbidities after accounting for age at assessment (*r* = 0.12, *p* = 0.21). Similarly, disease duration was not correlated with the number of vascular comorbidities after accounting for age at assessment (*r* = −0.12, *p* =0.21).

### Canonical Correlation Analysis

The canonical correlation analysis identified one statistically significant pair of variates (Pillai's trace = 0.45, *p* = 0.0035), which had a correlation of 0.50 (Supplementary Figure e3). Based on variable loadings, the biggest contributor to the cognitive variate was the SDMT (0.88), followed by verbal fluency (letter, 0.76), visual memory (0.52), verbal fluency (animals, 0.49); the CVLT-II verbal learning score was the smallest contributor (0.20) (Supplementary Figure e4). The biggest contributors to the MRI variate were gray matter MD (−0.79) and thalamic volume (0.63), followed by hippocampal volume (0.26) and NAWM MD (0.20) (Supplementary Figure e5). The canonical redundancy index for the cognitive variate (i.e., the total fraction of variance accounted for by the MRI variables) was 9.7%. The canonical redundancy index for the MRI variate was 7.2%. Age at MS symptom onset was not correlated with the cognitive variate (*r* = −0.13, *p* = 0.20).

After adjusting for disease-modifying therapy, vascular comorbidity was associated with the MRI variate (Table 2, global test χ^2^ = 16.88, *p* = 0.0007). We observed that the higher the number of vascular comorbidities, the lower the value of (i.e., the more abnormal) the MRI variate. Similarly, vascular comorbidity was associated with the cognitive variate (Table 2, global test χ^2^ = 9.78, *p* = 0.021) and we observed that the higher the number of vascular comorbidities the lower the value of the cognitive variate. After we added the MRI variate to the model, vascular comorbidity was no longer associated with the cognitive variate (global test χ^2^ = 2.0, *p* = 0.57) but the MRI variate was associated with the cognitive variate (χ^2^ = 22.98, *p* = <0.0001). Over one-third (37%) of the effect of vascular comorbidities on the cognitive variate was mediated by the MRI variate.

**Table 2:** Association of comorbidity with cognitive variate and magnetic resonance imaging (MRI) variate.

	**MRI variate**	**Cognitive variate^**b**^**	**Cognitive variate^**c**^**
	β (95% CI)^*^	β (SE)^*^	β (SE)^*^
**Vascular comorbidity** ^**a**^			
1	−0.54 (−0.94, −0.057)	−0.38 (−0.87, 0.11)	−0.096 (−0.58, 0.37)
	***p*** **= 0.015**	*p =* 0.12	*p =* 0.68
2	−0.76 (−1.35, −0.18)	−0.65 (−1.19, −0.040)	−0.32 (−0.84, 0.17)
	***p*** **=0.013**	***p*** **=0.025**	*p =* 0.23
≥3	−1.24 (−1.83, −0.50)	−0.91 (−1.52, −0.24)	−0.38 (−1.10, 0.38)
	***p*** **=0.0001**	***p*** **=0.05**	*p =* 0.32
Anxiety	0.036 (−0.56, 0.68)	0.48 (−0.17, 1.21)	0.43 (−0.18, 1.21)
	*p =* 0.91	*p =* 0.16	*p =* 0.21
Depression	0.60 (0.091, 1.18)	0.14 (−0.51, 0.77)	−0.12 (−0.73, 0.41)
	*p =* 0.03	*p =* 0.66	*p =* 0.67
Disease-modifying therapy	−0.082 (−0.45, 0.29)	−0.050 (−0.47, 0.34)	−0.029 (−0.40, 0.34)
	*p =* 0.68	*p =* 0.80	*p =* 0.88
MRI variate			0.46 (0.28, 0.64)
			***p*** **=0.0001**
Adjusted *R*^2^	0.12	0.045	0.23

* *Based on 1,000 bootstrap replications; a-reference group = 0; b-without adjustment for MRI variate; c-with adjustment for MRI variate. Bold indicates p < 0.05*.

### Multivariate Regression Analyses

In the multivariate regression analysis which included all cognitive variables as dependent variables, vascular comorbidity remained associated with cognition in the global test (χ^2^ = 26.9, *p* = 0.03). In the follow-up individual regression analyses, vascular comorbidity was associated with lower performance on the SDMT, CVLT-II, and verbal fluency (animal) (Table 3).

**Table 3:** Association of vascular comorbidity with individual cognitive tests.

	**SDMT**	**CVLT-II**	**BVMTR**	**COWAT-FAS**	**COWAT- Animals**
	β (SE)^*^	β (SE)^*^	β (SE)^*^	β (SE)^*^	β (SE)^*^
**Vascular comorbidity** ^**a**^					
1	−0.48 (−1.02, 0.030)	−0.63 (−1.13, −0.11)	−0.38 (−0.90, 0.14)	−0.30 (−0.82, 0.24)	−0.73 (−1.18, −0.29)
	*p =* 0.079	***p*** **= 0.018**	*p =* 0.15	*p =* 0.26	***p*** **= 0.001**
2	−0.61 (1–0.33, 0.13)	−0.52 (−1.23, 0.26)	−0.80 (−1.45, −0.11)	−0.47 (−1.06, 0.098)	−0.94 (−1.64, −0.35)
	*p =* 0.119	*p =* 0.20	***p*** **= 0.026**	*p =* 0.12	***p*** **= 0.004**
≥3	−0.94 (−1.74, −0.12)	−0.39 (−1.57, 0.51)	−0.55 (−1.45, 0.22)	−0.77 (−1.62, 0.037)	−0.87 (−1.65, 0.0055)
	***p*** **= 0.023**	*p =* 0.45	*P =* 0.20	*p =* 0.078	***p*** **= 0.037**
Global test vascular comorbidity	χ^2^ = 20.3, ***p*** **= 0.042**	χ^2^ = 22.9, ***p*** **= 0.018**	χ^2^ = 19.5, *p =* 0.052	χ^2^ = 19.6, *p =* 0.051	χ^2^ = 22.2, ***p*** **= 0.023**
Anxiety	0.45 (−0.42, 1.37)	0.071 (−1.05, 1.04)	0.43 (−0.73, 1.42)	0.049 (−0.69, 0.69)	(0.51, 1.78)
	*p =* 0.99	*p =* 0.90	*P =* 0.44	*p =* 0.89	***p*** **<0.0001**
Depression	−0.13 (−1.13, 0.76)	0.052 (−0.91, 0.81)	0.33 (−0.55, 1.23)	0.38 (−0.16, 0.88)	0.032 (−0.58, 0.17)
	*p =* 0.79	*p =* 0.90	*P =* 0.46	*p =* 0.16	*p =* 0.91
Disease-modifying therapy	−0.11 (−0.61, 0.32)	−0.27 (−0.032, 0.98)	−0.063 (−0.49, 0.46)	−0.12 (−0.63, 0.32)	−0.037 (−0.46, 0.35)
	*p =* 0.65	*p =* 0.29	*P =* 0.80	*p =* 0.63	*p =* 0.85

* *Based on 1,000 bootstrap replications; a- reference group = 0; SDMT, Symbol Digit Modalities Test; CVLT-II, California Verbal Learning Test-II; BVMT-R, Brief Visuospatial Memory Test-Revised; COWAT, Controlled Oral Word Association Test*.

*Bold indicates p < 0.05*.

In the multivariate regression analysis which included all MRI variables as dependent variables, the number of vascular comorbidities was associated with the MRI variables overall (χ^2^ = 39.7, *p* = 0.0001). In the follow-up individual regression analyses, the number of vascular comorbidities was associated was not associated with individual MRI measures (Table 4), indicating it was important to consider them in aggregate.

**Table 4:** Association of vascular comorbidity with individual magnetic resonance imaging measures.

	**Thalamic volume**	**Hippocampal volume**	**GM MD**	**NAWM MD**
	β (SE)^*^	β (SE)^*^	β (SE)^*^	β (SE)^*^
**Vascular comorbidity** ^**a**^				
1	−0.32 (0.39)	0.089 (0.27)	0.49 (0.32)	−0.48 (0.43)
	*p =* 0.41	*p =* 0.74	*p =* 0.13	*p =* 0.26
2	−0.52 (0.61)	0.23 (0.50)	0.70 (0.41)	−0.56 (0.60)
	*p =* 0.39	*p =* 0.65	*p =* 0.087	*p =* 0.35
≥3	−0.91 (0.59)	−0.28 (0.54)	0.86 (0.66)	−1.71 (0.70)
	*p =* 0.12	*P =* 0.60	*p =* 0.19	***p*** **= 0.015**
Global test vascular comorbidity	1.15	0.64	0.49	3.34
	*p =* 0.56	*p =* 0.72	*p =* 0.78	*p =* 0.19
Anxiety	−0.50 (0.43)	−0.23 (0.39)	0.049 (0.52)	0.53 (0.47)
	*P =* 0.24	*P =* 0.78	*p =* 0.92	*p =* 0.27
Depression	0.20 (0.50)	−0.013 (0.40)	−0.45 (0.44)	0.89 (0.57)
	0.69	*P =* 0.98	*p =* 0.31	*p =* 0.27
Disease-modifying therapy	−0.75 (0.36)	−0.079 (0.299)	0.35 (0.28)	0.99 (0.37)
	*P =* 0.039	0.78	*p =* 0.21	***p*** **= 0.007**

* *Based on 1,000 bootstrap replications; a- reference group = 0; GM, gray matter; MD, mean diffusivity; NAWM, normal-appearing white matter*.

*Bold indicates p < 0.05*.

Therefore, subsequent analyses focused on the association of vascular comorbidity with the SDMT, CVLT-II, and verbal (animal) fluency. After addition of the MRI variables to the model, vascular comorbidity was no longer associated with the cognitive variate in the global test (χ^2^ = 18.9, *p* = 0.22), nor with the individual cognitive variables SDMT (χ^2^ = 12.0, *p* = 0.36), CVLT-II (χ^2^ = 14.4, *p* = 0.21) or animal fluency (χ^2^ = 13.5, *p* = 0.26). Collectively, the MRI variables were associated with cognition in a global test (χ^2^ = 45.4, *p* = 0.001), and specifically with the SDMT (χ^2^ = 45.4, *p* = 0.001), the CVLT-II (χ^2^ = 28.5, *p* = 0.028) and animal fluency (χ^2^ = 28.3, *p* = 0.029).

### Complementary Analyses

After we limited our primary analyses to women, our findings were similar. Vascular comorbidity was associated with the cognitive variate in the model that did not include the MRI variate (χ^2^ = 18.8, *p* = 0.0003) but not when the MRI variate was added to the model (χ^2^ = 1.15, *p* = 0.76). After we included ever smoking in the count of vascular comorbidities, and repeated the primary analyses our findings were similar (Supplementary Table e5). When we limited the analysis to participants who were overweight or obese, our findings were similar (Supplementary Table e6).

## Discussion

In this cross-sectional study we assessed the inter-relationships between vascular comorbidity, brain structure as measured by MRI, and cognition among 105 individuals with MS enrolled from a population-based MS Clinic. We found that a higher number of vascular comorbidities was associated with lower cognitive function overall, and specifically with measures of processing speed, verbal learning and memory, and oral fluency. These associations were fully attenuated after we accounted for MRI measures of thalamic and hippocampal volume, and mean diffusivity of gray matter and NAWM, consistent with our hypothesis that the impacts of vascular comorbidity on cognition in people with MS are mediated by differences in brain structure (as depicted in Figure 1). This suggests that future intervention studies targeted at treating vascular comorbidity to improve cognition could use MRI measures as intermediate outcomes. It also highlights the complexity of relationships between comorbidity and outcomes in MS. Impacts of vascular comorbidity on brain health, including brain structure and cognition, may reflect increased peripheral inflammation, endothelial injury, and alterations in blood vessel function, cerebral blood flow and metabolism (69–72).

Some prior studies have reported an association between vascular comorbidities and brain volumes in persons with MS. The largest cross-sectional study to date, which included 6,409 from the MS-PATHS study, found that the presence of two or more vascular comorbidities was associated with lower whole brain and gray matter volumes (17). However, another MS-PATHS study including some of these participants, but based at a single center, found that while depression was associated with lower whole brain and gray matter volumes, hyperlipidemia was associated with higher whole brain volumes for unclear reasons (14). In the general population vascular comorbidities are also reportedly associated with differences in brain structure. A recent study including 9,722 participants from the UK Biobank found that the higher the total number of vascular risk factors the lower the brain volume and the greater the changes in brain microstructure (73). To our knowledge, prior studies in the MS population have not examined the association between vascular comorbidity and DTI measures. In the general population, vascular comorbidity is associated with differences in FA and MD in the NAWM. Higher systolic blood pressure and higher glucose in midlife are reportedly associated with worse white matter microstructure as measured using FA and MD (20, 74).

A handful of studies have examined the association between vascular comorbidity and cognition in people with MS. A study involving 11,506 individuals in the MS-PATHS study found that those with two or more vascular comorbidities, including diabetes, hypertension and hyperlipidemia, had lower correct scores on a test of processing speed, though no other cognitive domains were examined (17). A retrospective study involving 69 persons with MS found that a one-point increase in the Framingham risk score was associated with lower CVLT-II scores, and this appeared to be driven by male sex and higher lipid levels, though they did not observe any associations with the SDMT and BVMT-R (16). Even without overt cerebrovascular disease, hypertension, hypercholesterolemia, and diabetes are associated with cognitive impairment, an increased risk of dementia (75–77). However, a systematic review of several studies that included individuals without dementia reported that diabetes and hypertension were associated with reduced cognitive function (78). A 10-point increment in diastolic blood pressure (BP) is associated with increased odds of cognitive impairment (7%; 1–14%) in a North American sample even after controlling for numerous other factors (79). Thus, the findings reported for studies of MS samples appear consistent with these adverse impacts of vascular comorbidity, including diabetes and hypertension, on cognition in the general population.

Limitations to our study include our modest sample size, though we were careful to take several steps to reduce the number of variables examined and the number of comparisons performed in our primary analysis. Nonetheless, our findings should be replicated in other, larger populations. Most of our participants were women, consistent with the general female predominance of MS, thus our findings may not generalize as well to men with MS. While we did not comprehensively measure all cognitive domains, we assessed those most often affected in people with MS, those included in the BICAMS, and those affected by the comorbidities investigated here. Like other studies to date we were unable to account for the severity of vascular and other comorbidities or their treatments, and could not discriminate the effects of individual vascular comorbidities or behavioral factors such as smoking; this warrants further investigation. Prior studies have reported that depression and anxiety disorders are associated with lower cognitive performance and alterations in brain structure, and we did incorporate these variables into all of our regression models as covariates (80–82). Use of psychotropic medications may adversely influence cognition, however, their use overlapped substantially with the depression and anxiety disorders in our cohort, precluding an assessment of their effects (including whether they were mediated by changes in brain structure as illustrated in Figure 1 or via other pathways). Psychotropic medications may improve cognition as the psychiatric disorder remits, or worsen cognition (83). These effects on cognitive function may vary by drug class and possibly by specific agent, mandating the use of large samples to elucidate their effects. However, though we used the gold standard structured interview to identify these conditions, the small number of individuals affected precluded more detailed analysis. Our MRI protocol did not include gadolinium so it is possible that we included participants with focal inflammatory activity, which might have affected cognitive performance (84, 85). Other studies have suggested that vascular comorbidities, such as hyperlipidemia, are associated with an increase in gadolinium-enhancing lesions. Therefore, it is possible that a larger proportion of cognitive performance might have been mediated by MRI measures if we had been able to capture gadolinium-enhancing lesions. However, the proportion of participants with a relapse in the prior year was quite low. We used a small number of MRI measures to address multicollinearity and meet assumptions of our analyses. We focused on a subset of readily available MRI measures. Use of more advanced imaging measures, and incorporating other measures such as lesion volume might have increased the proportion of the vascular comorbidity effect on cognition mediated by MRI measures. That is, using a more limited set of measures may have biased our findings toward the null. Moreover, targeting more focal hypotheses may provide greater insight into the mechanisms evaluated herein. Although our study suggests that changes in MRI measures mediate the effects of vascular comorbidity on cognition, we cannot determine whether the changes in MRI measures solely reflect vascular effects similar to those in the general population, or whether the vascular comorbidities lead to increases in MS-specific pathologic changes. Finally, the cross-sectional nature of the study design limits causal inference. Nonetheless, cross-sectional studies that use mediation analyses can provide strong theoretical frameworks to guide future research, and more appropriately account for variables that lie in the same causal pathway than other approaches, as illustrated in the chronic pain literature (86). Future studies should examine these relationships longitudinally.

Our findings demonstrate that vascular comorbidity is associated with lower cognitive function in people with MS and this association is mediated, at least in part, via measurable changes in brain macrostructure and microstructure. This underscores the importance of preventing and treating vascular comorbidity effectively in persons with MS to mitigate their impacts on cognition and brain structure. Our findings highlight the importance of ensuring that etiologies other than MS, such as vascular comorbidity, are considered when evaluating individuals experiencing cognitive impairment. Our findings also suggest that additional MRI measures, such as DTI, may be considered useful methods of assessing the efficacy of interventions aimed at vascular comorbidities affecting persons with MS in the future, potentially warranting future consensus efforts (87).

## Data Availability Statement

The datasets presented in this article are not readily available because some participants did not agree to data sharing. Components of the datasets may be made accessible to qualified investigators with the appropriate ethical approvals and data use agreements upon request. Requests to access the datasets should be directed to RM, rmarrie@hsc.mb.ca.

## Ethics Statement

The studies involving human participants were reviewed and approved by University of Manitoba Health Research Ethics Board. The patients/participants provided their written informed consent to participate in this study.

## Author Contributions

RM: conceptualization, project administration, supervision, funding acquisition, and writing—original draft. JF: conceptualization, project administration, supervision, funding acquisition, and writing—review and editing. RP: conceptualization and writing—review and editing. CF: conceptualization, funding acquisition, methodology, and writing—review and editing. JK, JB, EM, JM, CB, and LG: conceptualization, funding acquisition, and writing—review and editing. CH: methodology, analysis, resources, and writing—review and editing. MU: methodology and writing—review and editing. TF: analysis and writing—review and editing. All authors contributed to the article and approved the submitted version.

## Funding

This study was funded by the Waugh Family Foundation MS Society of Canada Operating Grant (EGID 2639), CIHR (THC-135234), Crohn's and Colitis Canada, and the Waugh Family Chair in Multiple Sclerosis (to RM). CB was supported in part by the Bingham Chair in Gastroenterology.

## Conflict of Interest

RM receives research funding from: CIHR, Research Manitoba, Multiple Sclerosis Society of Canada, Multiple Sclerosis Scientific Foundation, Crohn's and Colitis Canada, National Multiple Sclerosis Society, CMSC, The Arthritis Society, US Department of Defense and UK MS Society. She serves on the Editorial Board of Neurology and Multiple Sclerosis Journal. She is a co-investigator on studies funded in part by Biogen Idec and Roche no funds to her or her institution. RP receives research funding from the Workers Compensation Board of Manitoba. CF receives research funding from the Brain Canada Foundation, MS Society of Canada, Natural Sciences and Engineering Research Council of Canada, Research Manitoba, and Health Sciences Centre Foundation and serves as Associate Editor of Frontiers in Neurology (Section on Applied Neuroimaging). JK receives research funding from the MS Society of Canada, University of Manitoba and Health Sciences Centre Foundation. JB receives research funding from CIHR, Brain and Behavior Research Foundation and the MS Society of Canada. LG receives research funding from CIHR, the MS Society of Canada and the Health Sciences Centre Foundation. She has consulted to Roche Canada. EM receives research funding from NSERC and St. Francis Xavier University, and other grant funding from the Council of Atlantic University Libraries. JM has conducted trials for Biogen Idec and Roche, and receives research funding from the MS Society of Canada. CB has consulted to Abbvie Canada, Amgen Canada, Bristol Myers Squibb Canada, Janssen Canada, Pfizer Canada, Roche Canada, Sandoz Canada, Takeda Canada, Mylan Pharmaceuticals, and Avir Pharmaceuticals. He has received unrestricted educational grants from Abbvie Canada, Janssen Canada, Pfizer Canada, Takeda Canada and Bristol Myers Squibb Canada. He has received investigator initiated grants from Abbvie Canada, Pfizer Canada, Amgen Canada, and Sandoz Canada. He has been on speaker's bureau of Abbvie Canada, Janssen Canada, Pfizer Canada, and Takeda Canada. JF receives research grant support from the Canadian Institutes of Health Research, the National Multiple Sclerosis Society, the Multiple Sclerosis Society of Canada, Crohn's and Colitis Canada, Research Nova Scotia; consultation and distribution royalties from MAPI Research Trust. The remaining authors declare that the research was conducted in the absence of any commercial or financial relationships that could be construed as a potential conflict of interest.

## Publisher's Note

All claims expressed in this article are solely those of the authors and do not necessarily represent those of their affiliated organizations, or those of the publisher, the editors and the reviewers. Any product that may be evaluated in this article, or claim that may be made by its manufacturer, is not guaranteed or endorsed by the publisher.
