# Supplementary material for: Effects of Vascular Comorbidity on Cognition in Multiple Sclerosis Are Partially Mediated by Changes in Brain Structure
Source: Front Neurol. 2022 May 24;13:910014. doi: 10.3389/fneur.2022.910014 (PMC9170886; doi:10.3389/fneur.2022.910014)
Supplement: Supplementary file 1 [file Data_Sheet_1.docx]

Supplementary Material

**Table e1. Scan Parameters**

| **Scan** | **Parameters** |
| --- | --- |
| T1w | repetition time [TR] = 1900 ms, echo time [TE] = 3.46 ms, inversion time [TI] = 900 ms, flip angle = 9˚, GRAPPA = 2, matrix size = 256 × 256, Field of View [FOV] = 250 × 250 mm^2^, number of slices = 176, slice thickness = 0.98 mm, number of averages = 1, band width [BW] = 170 Hz/Px, echo spacing [ESP] = 8.4 ms, acquisition time [TA] = 4:26 min |
| PD/T2w | TR = 4500 ms, TE1/TE2 (PD/T2w) = 11/101 ms, flip angle = 90˚, refocusing angle = 150˚, matrix size = 256 × 192, FOV = 250 × 187.5 mm^2^, number of slices = 54; slice thickness = 3.00 mm, BW= 250 Hz/Px, turbo factor = 6, number of averages = 1, spatial resolution = 0.98 × 0.98 × 3.00 mm^3^, TA = 3:45 min |
| FLAIR | TR = 9000 ms, TE = 100 ms, TI = 2499 ms, flip angle = 90˚, refocusing angle = 130˚, matrix size = 256 × 256, FOV = 240 × 240 mm^2^, number of slices = 32, slice thickness = 4.00 mm, BW = 287 Hz/Px, ESP = 7.17 ms, turbo factor = 16, BW = 287 Hz/Px, number of averages = 1, spatial resolution = 0.94 × 0.94 × 4.00 mm^3^, TA = 5:06 min |
| HARDI | TR = 3284 ms, TE = 89.4 ms, flip angle = 90°, refocusing angle = 177°, matrix size = 106 × 102, FOV = 212 × 204 mm^2^, number of slices = 80, slice thickness = 2.00 mm, MB factor = 4, number of averages = 1, BW = 1814 Hz/Px, ESP = 0.69 ms, phase partial Fourier = 6/8, spatial resolution = 2.00 × 2.00 × 2.00 mm^3^, TA = 6:34 min. This sequence was acquired twice, with opposite phase encoding directions – i.e., anterior-posterior (AP) and then posterior-anterior (PA) – for a total of 100 diffusion-weighted images (b=1500 s/mm^2^), 10 reference images (b=0 s/mm^2^), and an overall TA = 13:08 min |

**Table e2.** Characteristics of healthy controls used to develop regression-based norms

| **Characteristic** |  |
| --- | --- |
| **N** | 103 |
| **Age (year),** mean (SD) | 38.7 (16.3) |
| **Female**, n (%) | 68 (66.0) |
| **White**, n (%) | 85 (82.5) |
| **Years of education**, mean (SD) | 16.7 (3.0) |
| **Ever Smoker**, n (%) | 13 (12.6) |
| **BMI** (kg/m^2^), mean (SD) | 25.5 (4.7) |

**Table e3. Spearman correlations (p-values) between magnetic resonance imaging measures**

|  | **Whole Brain**  **Volume** | **Thalamus**  **Volume** | **Hippocampus**  **Volume** | **GM volume** | **WM volume** | **NAWM MD** | **WM MD** | **GM MD** |
| --- | --- | --- | --- | --- | --- | --- | --- | --- |
| **Whole Brain**  **Volume** | 1 | 0.78 | 0.65 | 0.85 | 0.83 | -0.26 | -0.51 | -0.73 |
|  |  | <0.0001 | <0.0001 | <0.0001 | <0.0001 | 0.0082 | <0.0001 | <0.0001 |
| **Thalamus volume** | 0.78 | 1 | 0.60 | 0.68 | 0.72 | -0.29 | -0.56 | -0.69 |
|  | <0.0001 |  | <0.0001 | <0.0001 | <0.0001 | 0.0026 | <0.0001 | <0.0001 |
| **Hippocampus Volume** | 0.65 | 0.60 | 1 | 0.58 | 0.56 | -0.17 | -0.30 | -0.42 |
|  | <0.0001 | <0.0001 |  | <0.0001 | <0.0001 | 0.091 | 0.0018 | <0.0001 |
| **GM Volume** | 0.85 | 0.68 | 0.58 | 1 | 0.49 | -0.31 | -0.44 | -0.65 |
|  | <0.0001 | <0.0001 | <0.0001 |  | <0.0001 | 0.0015 | <0.0001 | <0.0001 |
| **WM Volume** | 0.83 | 0.72 | 0.56 | 0.49 | 1 | -0.13 | -0.55 | -0.67 |
|  | <0.0001 | <0.0001 | <0.0001 | <0.0001 |  | 0.18 | <0.0001 | <0.0001 |
| **NAWM MD** | -0.26 | -0.29 | -0.17 | -0.31 | -0.13 | 1 | 0.55 | 0.33 |
|  | 0.0082 | 0.0026 | 0.091 | 0.0015 | 0.18 |  | <0.0001 | 0.0005 |
| **WM MD** | -0.51 | -0.56 | -0.30 | -0.44 | -0.55 | 0.5505 | 1 | 0.71 |
|  | <0.0001 | <0.0001 | 0.0018 | <0.0001 | <0.0001 | <0.0001 |  | <0.0001 |
| **GM MD** | -0.73 | -0.69 | -0.42 | -0.65 | -0.67 | 0.33 | 0.71 | 1 |
|  | <0.0001 | <0.0001 | <0.0001 | <0.0001 | <0.0001 | 0.0005 | <0.0001 |  |
|  |  |  |  |  |  |  |  |  |

GM = gray matter, WM = white matter, MD = mean diffusivity

**Table e4. Tests of multivariate normality**

| **Variables** | **Doornik-Hansen Test for Multivariate Normalit**y |
| --- | --- |
| Thalamic volume, hippocampal volume, MD NAWM, MD GM | χ2 = 14.0, p = 0.17 |
| SDMT, CVLT-II, BVMT-R, Verbal fluency | χ2 = 11.1, p = 0.35 |

GM = gray matter, NAWM = normal appearing white matter, MD = mean diffusivity, SDMT = Symbol Digit Modalities Test, CVLT-II = California Verbal Learning Test, BVMT-R = Brief Visuospatial Memory Test-Revised

**Table e5. Association of comorbidity with cognitive variate and magnetic resonance imaging (MRI) variate when smoking was incorporated into the vascular comorbidity count**

|  | **MRI variate** | **Cognitive variate^b^** | **Cognitive variate^c^** |
| --- | --- | --- | --- |
|  | **β (95% CI)*** | **β (SE)*** | **β (SE)*** |
| Vascular comorbidity^a^ |  |  |  |
| 1 | -0.23 (-0.67, 0.22)  p = 0.32 | -0.38 (-0.95, 0.17)  p = 0.18 | -0.24 (-0.81, 0.33)  p = 0.41 |
| 2 | -0.95 (-1.45, -0.45)  **p = 0.0001** | -0.87 (-1.43, -0.31)  **p = 0.002** | -0.43 (-1.06, 0.20)  p = 0.18 |
| ≥3 | -0.85 (-1.44, -0.27)  **p = 0.004** | -0.79 (-1.39, -0.19)  **p = 0.01** | -0.44 (-1.09, 0.20)  p = 0.17 |
| Anxiety | 0.056 (-0.47, 0.58),  p = 0.84 | 0.52 (-0.047, 1.10)  p = 0.07 | 0.48 (-0.12, 1.09)  p = 0.12 |
| Depression | 0.43 (-0.044, 0.90)  p = 0.075 | 0.028 (-0.52, 0.57)  p = 0.92 | -0.16 (-0.68, 0.36)  p = 0.55 |
| Disease-modifying therapy | 0.042 (-0.33, 0.41)  p = 0.83 | 0.029 (-0.34, 0.39)  p = 0.92 | -0.010 (-0.36, 0.33)  p = 0.95 |
| MRI variate |  |  | 0.45 (0.26, 0.63)  **p = 0.0001** |
| Adjusted R^2^ | 0.14 | 0.13 | 0.24 |

*Based on 1000 bootstrap replications; a- reference group = 0; b-without adjustment for MRI variate; c- with adjustment for MRI variate; 23.6% effect of vascular comorbidity on cognition mediated by MRI variables

**Table e6. Association of comorbidity with cognitive variate and magnetic resonance imaging (MRI) variate among individuals who were overweight or obese**

|  | **MRI variate** | **Cognitive variate^b^** | **Cognitive variate^c^** |
| --- | --- | --- | --- |
|  | **β (95% CI)*** | **β (SE)*** | **β (SE)*** |
| Vascular comorbidity^a^ |  |  |  |
| 1 | -0.54 (-1.06, -0.24)  **p = 0.04** | -0.54 (-1.06, -0.024)  **p = 0.04** | -0.30 (-0.82, 0.22)  p = 0.26 |
| 2 | -0.74 (-1.45, -0.023)  **p = 0.043** | -0.74 (-1.45, -0.023)  **p = 0.043** | -0.53 (-1.16, 0.097)  p = 0.18 |
| ≥3 | -1.42 (-2.03, -0.81)  **p = 0.0001** | -0.1.42 (-2.03, -0.81)  **p = 0.0001** | -0.88 (-1.60, -0.16)  **p = 0.017** |
| Anxiety | 0.39 (-0.30, 1.09),  p = 0.27 | 0.39 (-0.30, 1.08)  p = 0.27 | 0.31 (-0.42, 1.03)  p = 0.41 |
| Depression | 0.51 (-0.12, 1.15)  p = 0.11 | 0.51 (-0.12, 1.15)  p = 0.11 | 0.24 (-0.31, 0.79)  p = 0.40 |
| Disease-modifying therapy | -0.14 (-0.58, 0.30)  p = 0.54 | -0.14 (-0.58, 0.30)  p = 0.54 | -0.056 (-0.47, 0.36)  p = 0.79 |
| MRI variate |  |  | 0.44 (0.26, 0.63)  **p = 0.0001** |
| Adjusted R^2^ | 0.11 | 0.11 | 0.28 |

*Based on 1000 bootstrap replications; a- reference group = 0; b-without adjustment for MRI variate; c- with adjustment for MRI variate

26% effect of comorbidity on cognition mediated via MRI variables

Figure e1. Scatterplots of MRI variable z-scores against each other


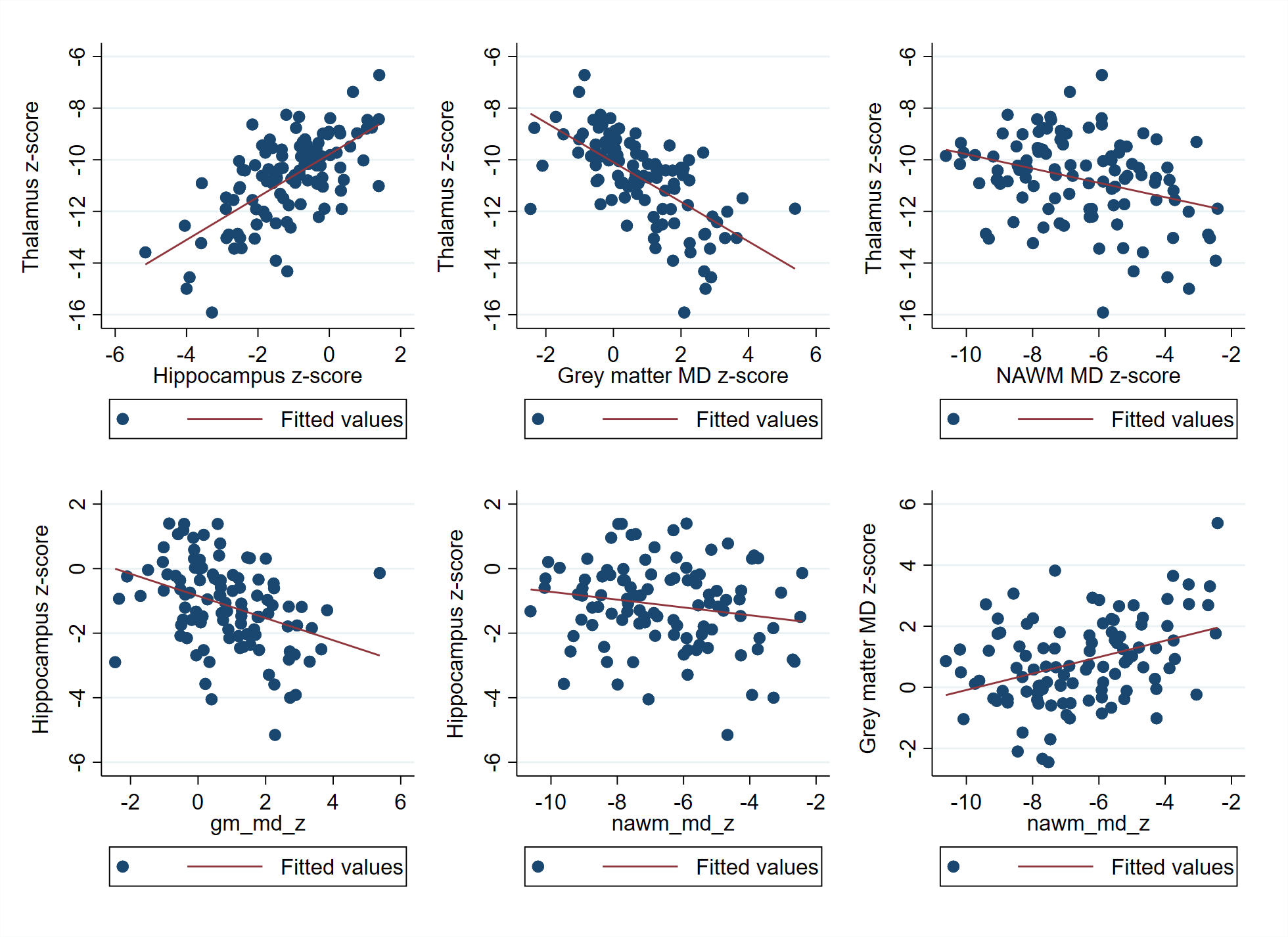


Figure e2. Scatterplots of cognitive test z-scores against each other


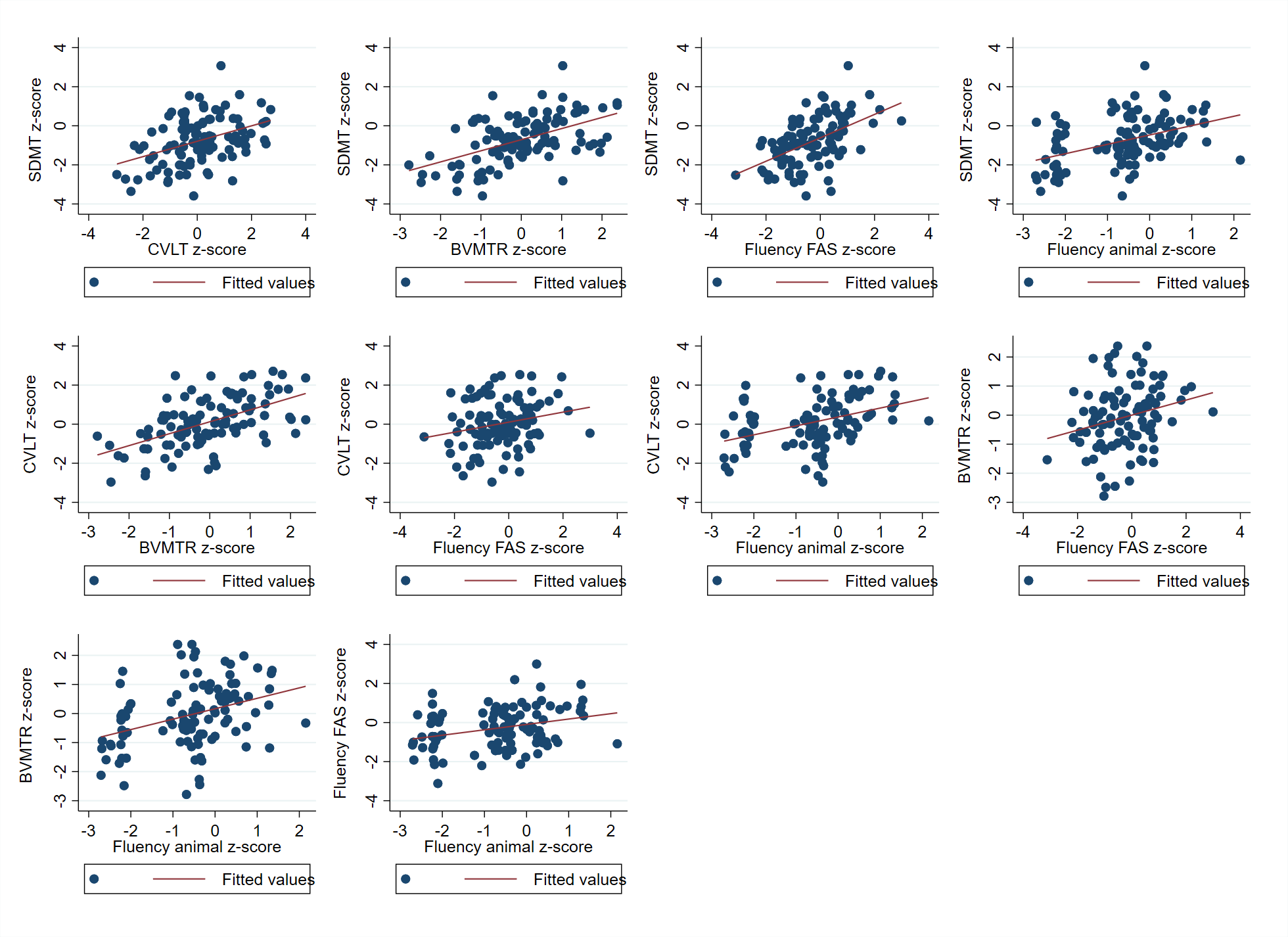


SDMT = Symbol Digit Modalities Test; CVLT-II = California Verbal Learning Test-II; BVMT-R = Brief Visuospatial Memory Test-Revised; COWAT = Controlled Oral Word Association Test/Fluency

Figure e3. Scatterplot of the association between the cognitive variate and the MRI variate


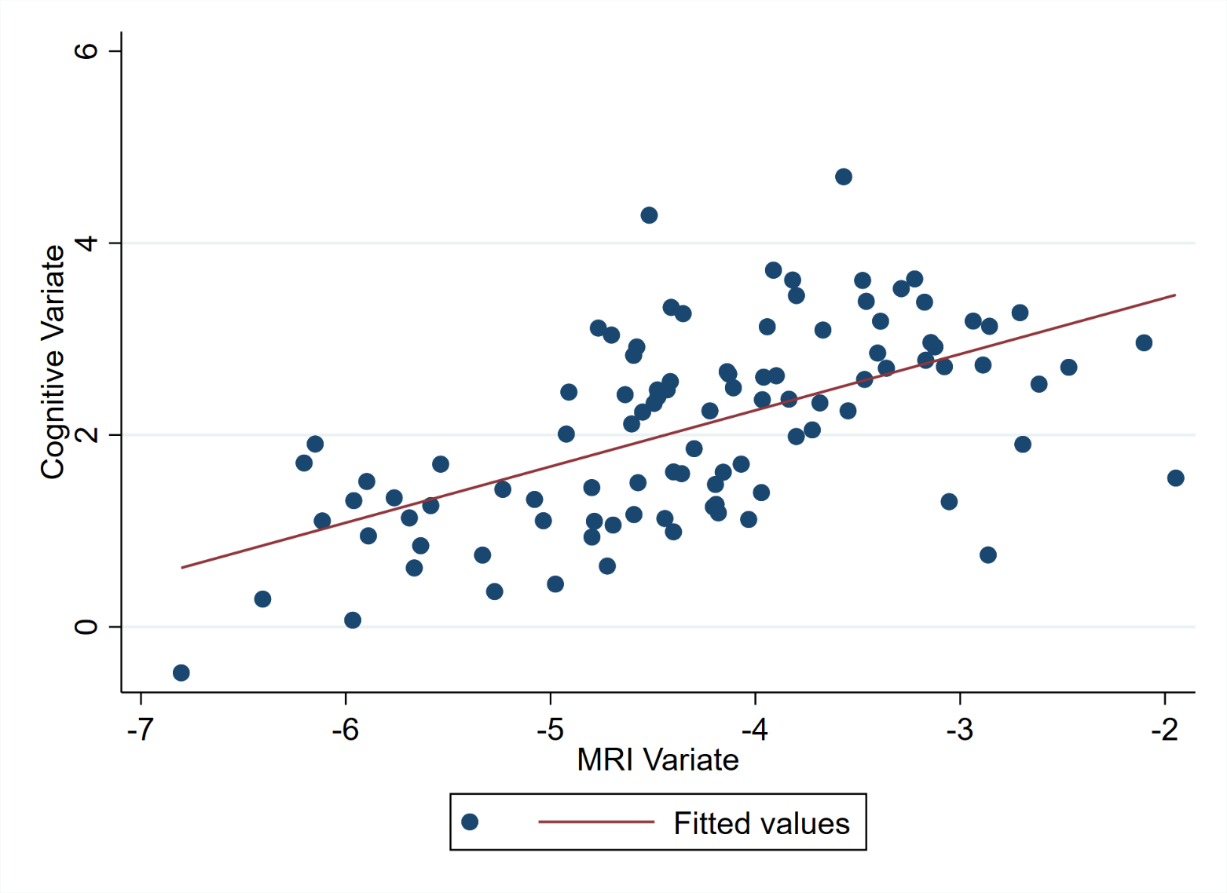


Figure e4. Scatterplots of cognitive test z-scores versus the cognitive variate


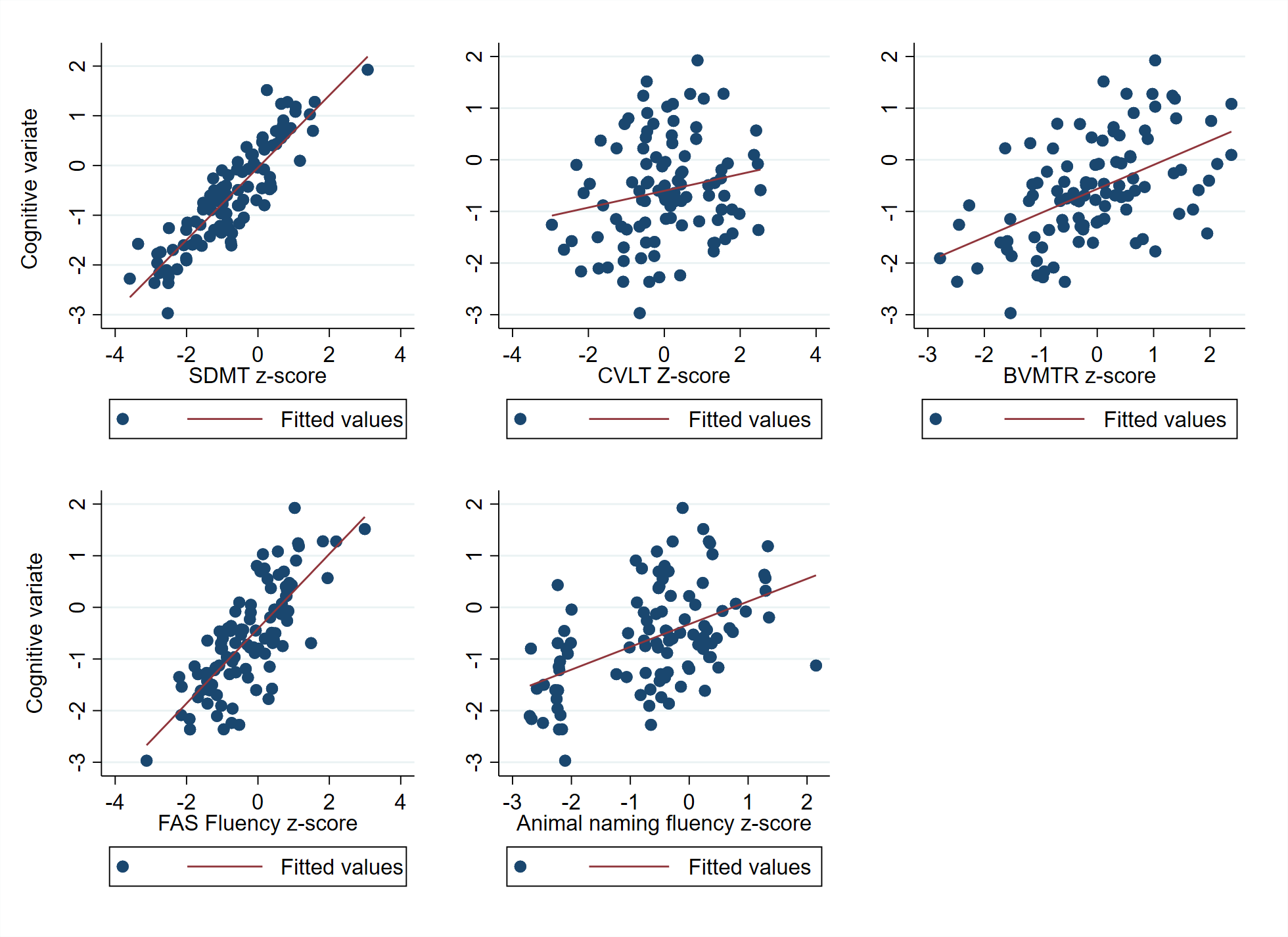


SDMT = Symbol Digit Modalities Test; CVLT-II = California Verbal Learning Test-II; BVMT-R = Brief Visuospatial Memory Test-Revised; COWAT = Controlled Oral Word Association Test/Fluency

Figure e5. Scatterplots of MRI variable z-scores versus the MRI variate


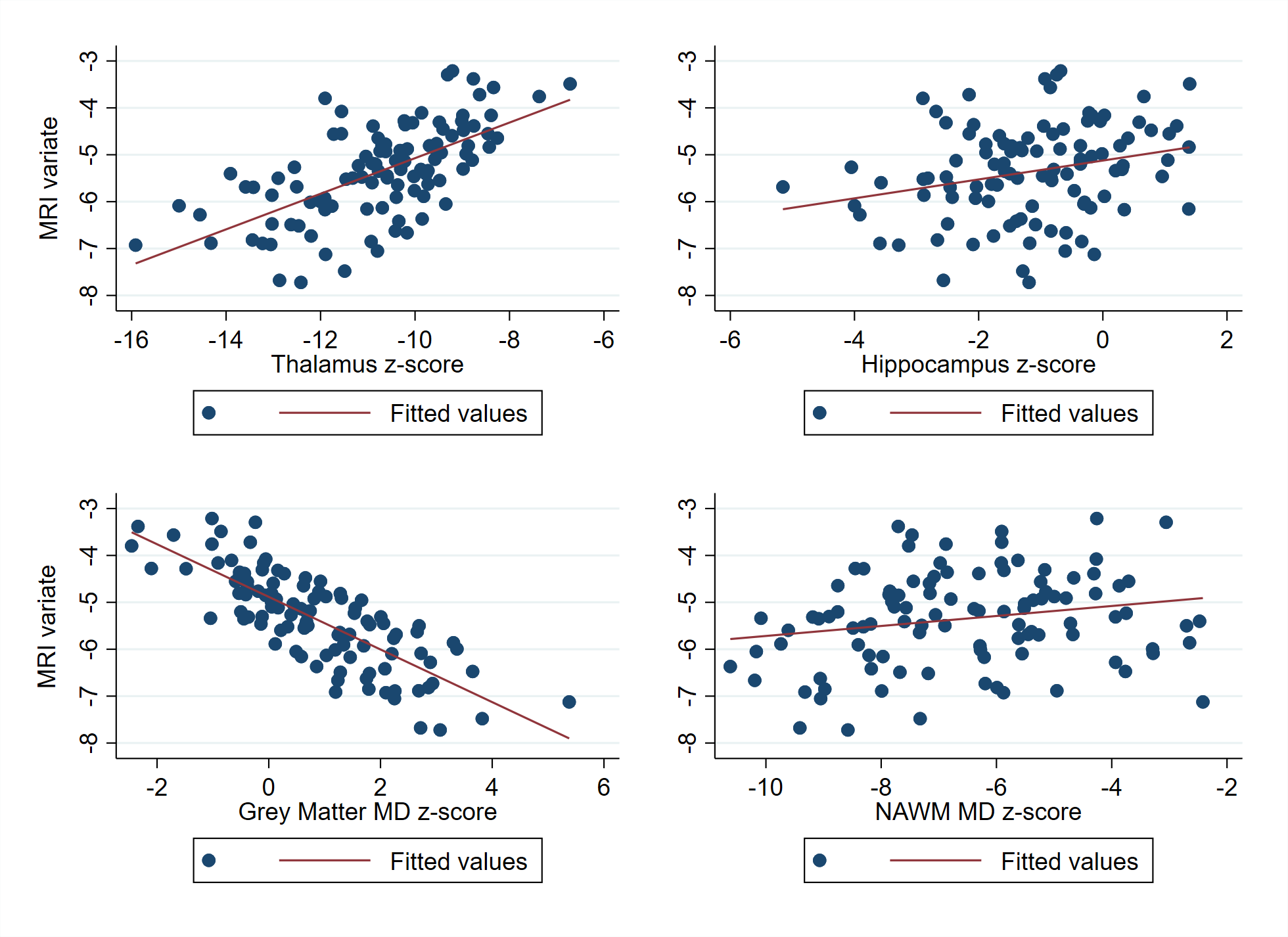


**
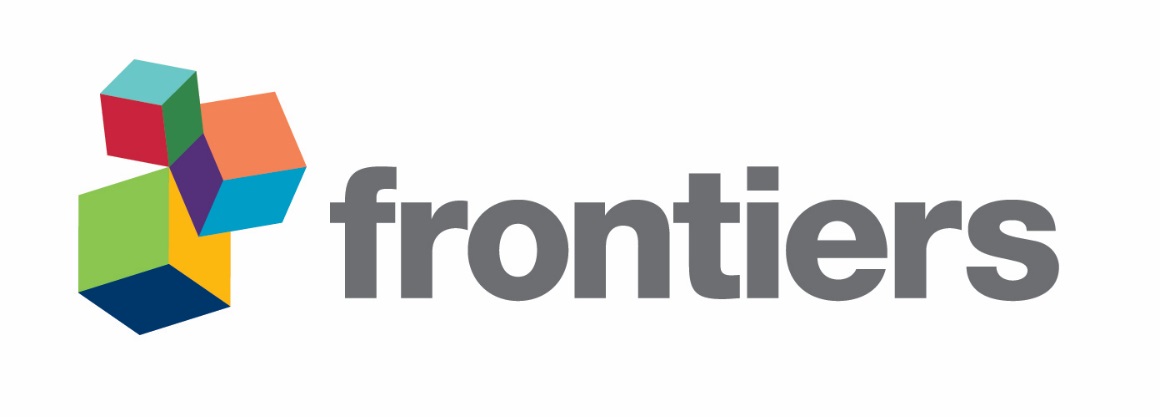
**
